# High-resolution EEG dataset during resting and localized visual stimulation in the lower-left visual field

**DOI:** 10.1016/j.dib.2025.112400

**Published:** 2025-12-17

**Authors:** Takayuki Onojima, Keiichi Kitajo

**Affiliations:** a Rhythm-based Brain Information Processing Unit, CBS-TOYOTA Collaboration Center, RIKEN Center for Brain Science, Japan; b Data Science and AI Innovation Research Promotion Center, Shiga University, Japan; c Center for Training Professors in Statistics, Institute of Statistical Mathematics, Japan; d Division of Neural Dynamics, Department of System Neuroscience, National Institute for Physiological Sciences, National Institutes of Natural Sciences, Japan; e Physiological Sciences Program, Graduate Institute for Advanced Studies, SOKENDAI, Japan

**Keywords:** High-sampling EEG, Visual perception, Visual evoked potentials, Checkerboard stimulation, Brain imaging data structure, Open data

## Abstract

This dataset provides high–temporal–resolution electroencephalography (EEG) recordings collected from eight healthy adults during two conditions: (1) eyes-open resting state and (2) localized visual stimulation using a static, sector-shaped checkerboard presented in the lower-left visual field. EEG was recorded at 10,000 Hz in DC mode using a 64-channel actiCAP slim system (BrainProducts GmbH) and Bittium NeurOne Tesla amplifiers. All recordings are organized according to the Brain Imaging Data Structure (BIDS v1.9.0) and distributed as raw BrainVision files with complete metadata. The dataset includes behavioral responses for target-dot detection and detailed event annotations for each contrast level. Example preprocessing and analysis scripts are provided in MNE-Python to facilitate reproducible event-related potentials (ERPs) and time–frequency analyses. These high-sampling EEG recordings allow investigation of early visual evoked potentials, oscillatory phase alignment, and hemispheric lateralization under localized visual stimulation.

Specifications Table

**Table:** 

Subject	Health Sciences, Medical Sciences & Pharmacology
Specific subject area	EEG, visual perception, electrophysiology, data standards
Type of data	Raw EEG signals, Event markers, Channel information, Metadata (JSON/TSV)
Data collection	EEG data were collected from eight healthy adults using an actiCAP slim electrode system (BrainProducts GmbH, Germany) connected to two 32-channel Tesla amplifiers (Bittium NeurOne, Finland) operated in DC mode at 10,000 Hz with 24-bit resolution. A total of 67 channels were recorded (63 scalp electrodes, HEOG/VEOG, right earlobe, photodiode). Visual stimuli were presented on a 144 Hz LCD monitor as static, sector-shaped checkerboard patches at four luminance-contrast levels with occasional target-dot trials. Resting-state data (eyes open, 4 min) were also obtained for comparison. All recordings were referenced to the left earlobe and grounded at AFz.
Data source location	RIKEN Center for Brain Science, Wako, Saitama, Japan
Data accessibility	Repository name: CBS Data Sharing Platform, RIKEN Center for Brain Science, Wako, Saitama, Japan Data identification number: 10.60178/cbs.20251213-001 Direct URL to data: https://neurodata.riken.jp/id/20251213-001 Instructions for accessing these data (for peer review only): The dataset is currently available in a finalized form on the RIKEN platform, but access is password-protected during peer review. Editors and reviewers may access the full dataset using the following credentials: Password: jU2 %D#n6MriP No account registration or personal information is required to access the data. Upon acceptance, the data will be made publicly available under the CC BY-NC 4.0 license.
Related research article	None(dataset article only)

## 1 Value of the Data

• The EEG was recorded at a high sampling rate (10 kHz), providing sufficient temporal resolution for precise synchronization with stimulus timing.
• The high temporal resolution facilitates reliable single-trial analysis, which is particularly advantageous for closed-loop experiments and brain–machine interface (BMI) applications that require real-time decoding of brain activity.
• The localized visual stimulation in the lower-left visual field minimizes foveal interference and elicits contralateral dorsal occipital responses, offering a well-defined spatially specific visual input.
• Each participant was assigned 200 trials for each of the four luminance-contrast conditions, providing a rich dataset for statistical modeling, single-trial analysis, and algorithm development.
• The timing of stimulus presentation was recorded using both digital triggers and a photodiode sensor, ensuring sub-millisecond precision.
• The dataset supports research on visual evoked potentials, oscillatory phase alignment, and the development and validation of real-time EEG analysis or decoding methods.

## 2 Background

EEG provides high temporal resolution for studying visual information processing, particularly in response to structured visual stimuli such as checkerboard patterns. The present dataset was originally recorded to investigate the relationship between the ongoing brain state at the moment of visual stimulus presentation and the corresponding neural responses. Although high sampling rates were not the primary focus of the study, it was necessary to record EEG at a relatively high temporal resolution to accurately capture both the pre-stimulus brain state and the subsequent evoked responses. Eight healthy participants completed the experiment in December 2019. Further data collection was discontinued due to the global COVID-19 pandemic. To enhance usability and reproducibility, the dataset has been released as an independent open-access resource. The data consist of high-resolution EEG recordings obtained during localized, sector-shaped checkerboard stimulation presented in the lower-left visual field and are organized according to the EEG-BIDS standard.

## 3 Data Description

The dataset includes EEG recordings from eight healthy adults (aged 20–30 years; four males and four females) under two conditions: eyes-open resting state and localized visual stimulation. All participants were right-handed, had normal or corrected-to-normal vision, and reported no history of neurological or ophthalmological disorders. EEG was recorded with an actiCAP slim electrode system (BrainProducts GmbH, Germany) connected to two 32-channel Tesla EEG amplifiers (Bittium NeurOne, Finland). A total of 67 channels were recorded using the NeurOne EEG system, comprising 64 EEG electrodes from the actiCAP system positioned according to the international 10–10 layout (including the right earlobe), two bipolar electrooculogram (EOG) channels for horizontal and vertical eye movements, and one photodiode channel used to record changes in screen luminance for precise stimulus timing. The signals were recorded in DC mode at 10,000 Hz with 24-bit resolution. Data were referenced online to the left earlobe, and an averaged earlobe reference (left + right) was computed during preprocessing. The dataset is organized according to the EEG-BIDS v1.9.0 standard [1,2], following a consistent directory hierarchy across all participants (sub-01 – sub-08).

Each participant folder contains two main subdirectories:

beh/ for behavioral response data and eeg/ for raw EEG recordings with accompanying metadata.

A representative example (sub-01) is shown below:

Each run file corresponds to one recording block of the visual stimulation task, while the behavioral .tsv files store reaction times for target trials. EEG metadata in JSON and TSV sidecars describe channel locations, event timing, and acquisition parameters, enabling reproducible preprocessing and analysis. For detailed experimental setup and stimulus design, see the next section.

## 4 Experimental Design, Materials and Methods

### 4.1 Experimental setup

Participants were seated in a dark, electrically shielded room, approximately 1 m from an LCD monitor (BenQ XL2420, 144 Hz, 1920 × 1080 pixels). The luminance of the display was measured with a photometer before the experiment. The white, gray, and black levels of the monitor were approximately 153, 79, and 8 cd/m², respectively. The display was adjusted so that the output luminance changed as linearly as possible from white to black. A chin rest was used to stabilize head position and maintain viewing distance. Visual stimuli were generated using MATLAB (MathWorks) with Psychtoolbox, presented on a uniform gray background.

### 4.2 Conditions

**Fig. 1:**
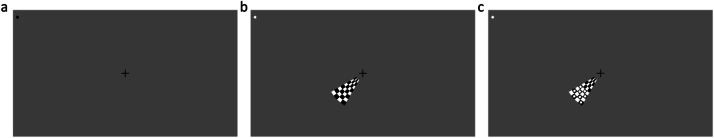
Visual display conditions: (a) fixation screen, (b) non-target checkerboard stimulus, and (c) target checkerboard stimulus with small white dots. All stimuli were presented on a uniform gray background at a viewing distance of 1 m. The small black or white circle shown at the upper-left corner of each panel indicates the position of the photodiode sensor used for precise stimulus timing. The sensor was placed directly over this circle, which appeared white during stimulus presentation and black otherwise. The figure shows the high-contrast condition for both target and non-target stimuli; images for other contrast levels are available in the stimuli folder of the shared dataset.

Resting state: 4-minute eyes-open fixation on a uniform gray background (Fig. 1a).

Localized Visual stimulation: A lower-left sector-shaped checkerboard was intermittently presented on a gray background.

### 4.3 Localized Visual stimulation task

The stimulus consisted of a sector-shaped checkerboard patch presented in the lower-left visual field (Fig. 1b). A small central fixation cross (< 2° in radius) was continuously visible at the center. The checkerboard occupied an annular region between 2° and 10° of eccentricity (visual angle from the fixation point) and extended approximately 30° in polar angle (120°–150°, centered at ∼135°), defined in polar coordinates centered on the fixation point, where 0° corresponds to the right horizontal meridian (Fig. 1b). The checkerboard comprised five radial and twenty-four angular cycles of alternating black and white checks. Each stimulus was displayed for a randomly selected duration between 1.0–1.5 s and was followed by a gray background. The stimuli were static (non-flickering) checkerboard patches presented intermittently, designed to evoke transient visual evoked potentials (VEPs) rather than steady-state responses. A small BrainProducts photo sensor (BrainProducts GmbH, Germany) was attached to the upper-left corner of the display, positioned over a circular patch that appeared white during stimulus presentation and black otherwise, providing an independent measure of screen luminance changes for precise stimulus timing. Participants were instructed to maintain fixation throughout the experiment.

The visual stimulation task included four contrast conditions to examine responses to different levels of luminance contrast. Each condition consisted of both target and non-target stimuli, which were presented in a pseudorandom order within each run. Four contrast levels were defined by varying the luminance difference between the white and black checks (w–b) while keeping the background luminance fixed at gray = 0.5 (normalized units; black = 0, white = 1). The luminance differences (w–b) were set to 1, 1/4, 1/16, and 1/64 for the high-, medium-high-, medium-low-, and low-contrast conditions, respectively. Accordingly, the black and white values were symmetrically adjusted around the gray background:

0 and 1 for the high-contrast condition (w–*b* = 1),

3/8 and 5/8 for medium-high (w–*b* = 1/4),

15/32 and 17/32 for medium-low (w–*b* = 1/16),

and 63/128 and 65/128 for the low-contrast condition (w–*b* = 1/64).

This design maintained a constant mean luminance across all contrast levels while systematically reducing the contrast, producing four distinct visual intensities. In 20 % of trials (target condition, Fig. 1c), small white dots were superimposed on the checkerboard, and participants responded with a mouse click. The remaining 80 % (non-target, Fig. 1b) contained no dots. Each run included 200 trials (160 non-target and 40 target), evenly distributed across the four contrast conditions (40 non-target and 10 target trials per contrast). The experiment consisted of five runs (∼8 min each) with 1–2 min breaks between runs to minimize fatigue, resulting in a total of 200 non-target and 50 target trials per contrast level across the session.

The geometry of the stimulation was chosen to avoid foveal interference and to evoke localized responses in the visual cortex, particularly in dorsal occipital regions representing the lower visual field. Stimulation was restricted to the lower-left visual field to accommodate four luminance-contrast conditions while maintaining sufficient trials per condition and keeping the total experiment duration within a reasonable time frame. This configuration was optimized for method development, prioritizing a larger number of trials using a single, well-defined stimulus location rather than testing multiple visual quadrants.

### 4.4 EEG recording setup

EEG was recorded using an actiCAP slim (BrainProducts GmbH, Germany) connected to two Tesla EEG amplifiers (Bittium NeurOne, Finland), operated in DC mode at 10,000 Hz with 24-bit resolution. The online low-cut frequency was DC (0 Hz), and the high-cut filter was set to 3600 Hz during acquisition. All recordings were referenced to the left earlobe and grounded at AFz. Electrode impedances were maintained below 10 kΩ throughout all recordings.

A total of 67 channels were recorded:

63 scalp electrodes (10–10 system)

64: right earlobe (used for averaged reference)

65–66: horizontal (HEOG) and vertical (VEOG) electrooculograms.

67: photodiode sensor recording screen luminance changes.

The sensor was attached to the upper-left corner of the display, positioned over a small circular patch (white or black) presented in that location to indicate stimulus timing.

Triggers marking stimulus onset, offset, and target events were automatically logged in the EEG data stream.

### 4.5 Example preprocessing and analyses

Example scripts (MNE-Python) demonstrate dataset reusability [3].

Code availability:

The Python scripts used for example analyses are publicly available at:

https://github.com/Takayuki-Onojima/EEG-checkerboard-BIDS-examples

Continuous EEG data were re-referenced to linked earlobes (average of left and right) and resampled from 10 kHz to 1000 Hz. For VEP preprocessing, the data were band-pass filtered between 0.01 and 30 Hz. Ocular artifacts were corrected using ICA implemented in MNE-Python [3,4]. ICA was fitted on a high-passed (≥1 Hz) and downsampled (200 Hz) copy of the data to improve stability and computation speed. EOG-related components were automatically detected via correlation- and epoch-based methods (thresholds 2.0–3.0 SD) and removed from the original data. Channels corresponding to the right earlobe, photodiode, and noisy or unstable electrodes (I1, I2) were excluded from ICA fitting.

### 4.6 Evoked and time–frequency results

**Fig. 2:**
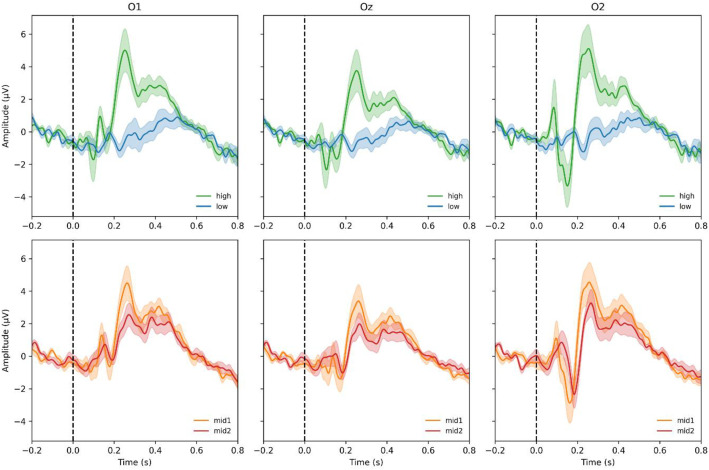
Grand-average evoked potentials (group mean ± SEM) recorded at posterior electrodes O1, Oz, and O2 for each stimulus intensity. Evoked responses were computed per subject from VEP-cleaned data and then averaged across eight participants (*N* = 8). Traces show the grand mean (solid lines) and standard error of the mean (shaded areas) for four stimulus intensities (high, mid1, mid2, low). Data were baseline-corrected to −0.2 to −0.05 s and plotted from −0.2 to 0.8 s relative to stimulus onset. The vertical dashed line at 0 s indicates stimulus onset (color coding: high = green, mid1 = orange, mid2 = red, low = blue).

ERP averaging revealed clear transient visual evoked potentials (VEPs) with P1/N1 components in posterior electrodes (O1, Oz, O2) around 100–150 ms post-stimulus [5,6]. Fig. 2 shows grand-average ERPs (mean ± SEM) across participants for the four contrast conditions (high, mid1, mid2, low).

**Fig. 3:**
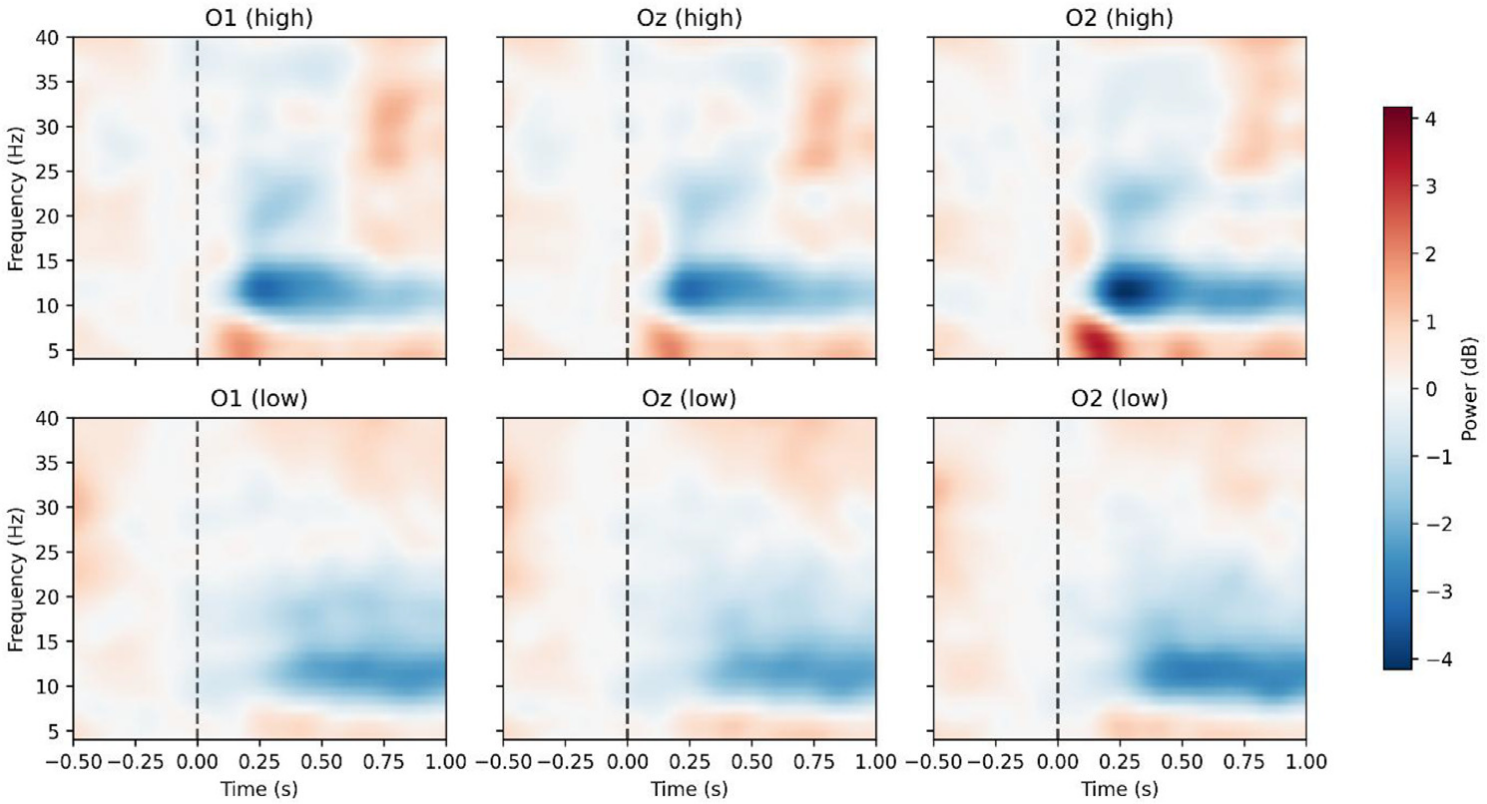
Group-level time–frequency representations (TFRs) of power (dB) at posterior channels O1, Oz, and O2 for stimulus intensities. TFRs were computed with complex Morlet wavelets (4–40 Hz, n_cycles = freq/2) and converted to decibels relative to a baseline interval of −0.20 to −0.05 s. Results were averaged across subjects (*N* = 8). A post-stimulus decrease in alpha-band power (8–13 Hz) is visible, particularly for the high-intensity condition. The dashed vertical line marks stimulus onset.

Time–frequency analysis (4–40 Hz; complex Morlet wavelets) revealed a clear post-stimulus α-power suppression (8–13 Hz), consistent with visual cortical activation [7]. Fig. 3 presents group-averaged time–frequency representations (TFRs) relative to baseline (−0.2 to −0.05 s), showing pronounced α-power reduction particularly for high-intensity stimuli.

## Limitations

The dataset includes EEG recordings from eight participants, which may limit generalization across a broader population. Each participant completed multiple sessions and runs, resulting in rich within-subject data suitable for testing preprocessing or analysis methods. Minor variations in electrode impedance and slow baseline drifts can occur and are visible in DC-coupled recordings, as low-frequency components are preserved without hardware filtering. No preprocessed data are provided to maintain neutrality for future users. Researchers are encouraged to apply their own preprocessing pipelines appropriate to their analytical goals.

## Ethics Statement

All participants provided written informed consent in accordance with the Declaration of Helsinki. The experimental protocol was approved by the RIKEN Center for Brain Science Ethics Committee (Wako3 26–24).

## CRediT Author Statement

Takayuki Onojima: Conceptualization, Data collection, Data curation, BIDS conversion, Formal analysis, Visualization, Resources, Funding acquisition, Writing – original draft.

Keiichi Kitajo: Resources, Funding acquisition, Writing – review & editing.

## Declaration of Generative AI and AI-Assisted Technologies in the Manuscript Preparation Process

During the preparation of this work, the authors used ChatGPT (OpenAI, GPT-5) to improve the clarity and grammar of English text. After using this tool, the authors reviewed and edited the content as needed and take full responsibility for the content of this publication.

## Data Availability

CBS Data Sharing PlatformHigh-resolution EEG dataset during resting and localized visual stimulation in the lower-left visual field (Original data).
